# Levodopa-Carbidopa Intestinal Gel in Advanced Parkinson'd Disease: Final 12-Month, Open-Label Results

**DOI:** 10.1002/mds.26123

**Published:** 2014-12-24

**Authors:** Hubert H Fernandez, David G Standaert, Robert A Hauser, Anthony E Lang, Victor SC Fung, Fabian Klostermann, Mark F Lew, Per Odin, Malcolm Steiger, Eduard Z Yakupov, Sylvain Chouinard, Oksana Suchowersky, Jordan Dubow, Coleen M Hall, Krai Chatamra, Weining Z Robieson, Janet A Benesh, Alberto J Espay

**Affiliations:** 1 Center for Neurological Restoration, Cleveland ClinicCleveland, Ohio, USA; 2 University of Alabama at BirminghamBirmingham, Alabama, USA; 3 Department of Neurology, University of South FloridaTampa, Florida, USA; 4 University of TorontoToronto, Ontario, Canada; 5 Movement Disorders Unit, Westmead Hospital and Sydney Medical SchoolSydney, Australia; 6 Charité-University Medicine BerlinBerlin, Germany; 7 Keck/USC School of MedicineLos Angeles, California, USA; 8 Klinikum-Bremerhaven, Bremerhaven, Germany and Skane University HospitalLund, Sweden; 9 Walton Center for Neurology and NeurosurgeryLiverpool, United Kingdom; 10 Kazan State Medical UniversityKazan, Russia; 11 University of MontrealMontreal, Quebec, Canada; 12 University of AlbertaEdmonton, Alberta, Canada; 13 AbbVie Inc., North ChicagoIllinois, USA; 14 University of Cincinnati Academic Health CenterCincinnati, Ohio, USA

**Keywords:** dyskinesia, infusion, levodopa-carbidopa intestinal gel, “off” time, percutaneous endoscopic gastrojejunostomy

## Abstract

Motor complications in Parkinson's disease (PD) are associated with long-term oral levodopa treatment and linked to pulsatile dopaminergic stimulation. l-dopa-carbidopa intestinal gel (LCIG) is delivered continuously by percutaneous endoscopic gastrojejunostomy tube (PEG-J), which reduces l-dopa-plasma–level fluctuations and can translate to reduced motor complications. We present final results of the largest international, prospective, 54-week, open-label LCIG study. PD patients with severe motor fluctuations (>3 h/day “off” time) despite optimized therapy received LCIG monotherapy. Additional PD medications were allowed >28 days post-LCIG initiation. Safety was the primary endpoint measured through adverse events (AEs), device complications, and number of completers. Secondary endpoints included diary-assessed off time, “on” time with/without troublesome dyskinesia, UPDRS, and health-related quality-of-life (HRQoL) outcomes. Of 354 enrolled patients, 324 (91.5%) received PEG-J and 272 (76.8%) completed the study. Most AEs were mild/moderate and transient; complication of device insertion (34.9%) was the most common. Twenty-seven (7.6%) patients withdrew because of AEs. Serious AEs occurred in 105 (32.4%), most commonly complication of device insertion (6.5%). Mean daily off time decreased by 4.4 h/65.6% (*P* < 0.001). On time without troublesome dyskinesia increased by 4.8 h/62.9% (*P* < 0.001); on time with troublesome dyskinesia decreased by 0.4 h/22.5% (*P* = 0.023). Improvements persisted from week 4 through study completion. UPDRS and HRQoL outcomes were also improved throughout. In the advanced PD population, LCIG's safety profile consisted primarily of AEs associated with the device/procedure, l-dopa/carbidopa, and advanced PD. LCIG was generally well tolerated and demonstrated clinically significant improvements in motor function, daily activities, and HRQoL sustained over 54 weeks. © 2014 The Authors. Movement Disorders published by Wiley Periodicals, Inc. on behalf of International Parkinson and Movement Disorder Society.

Oral levodopa (L-dopa) is one of the most effective therapies for Parkinson's disease (PD).^1^–^4^ During early disease stage, motor symptoms are well controlled with 3 to 4 daily doses. As PD progresses, however, oral l-dopa's effect may not be sustained between doses and symptoms may re-emerge.^3^–^6^ Adjunctive therapies may initially reduce the duration of motor complications (i.e., decrease “off” time by approximately 1-2 hours per day); however, dyskinesia and other adverse events (AEs) increase.^7^–^9^ Oral l-dopa pharmacokinetic properties contribute to oscillations in plasma l-dopa levels,^3^–^5^ which may be compounded by variability of gastric emptying and l-dopa absorption.^10^,^11^

Establishing stable plasma l-dopa levels may provide more continuous dopaminergic stimulation, resulting in decreased motor fluctuations.^4^,^6^ l-dopa-carbidopa intestinal gel (LCIG) offers continuous drug delivery and may provide a closer approximation of physiological continuous dopaminergic stimulation through its amelioration of plasma-level fluctuations, including the depth and frequency of serum troughs.^5^ LCIG is delivered continuously by portable pump through a percutaneous endoscopic gastrojejunostomy (PEG-J) tube,^1^,^6^,^12^ bypassing the stomach to eliminate variability associated with gastric emptying,^4^,^10^ resulting in a significant decrease in off time duration.^5^,^12^–^15^

The efficacy of LCIG as an adjunctive therapy was evaluated in a double-blind, double-dummy, phase III study, which showed a 4.0-hour reduction from baseline in off time among patients randomized to LCIG and a 1.9-hour difference in off time reduction versus optimized oral l-dopa (*P* = 0.0015).^15^ Previous open-label studies also showed statistically significant reductions in off time and/or dyskinesia versus baseline.^13^,^14^,^16^–^21^ However, these studies were small by design, comprised of 5 to 91 patients. This large study was designed to provide needed longer-term safety and efficacy results with clinical applicability to an international patient population with advanced PD. Furthermore, the study investigated the initiation and maintenance of LCIG as monotherapy, replacing adjunctive PD therapy.

## Patients and Methods

The safety and efficacy of LCIG were evaluated in patients with advanced PD experiencing motor fluctuations despite optimized medical therapy in an open-label, phase III, 12-month study (http://ClinicalTrials.gov: NCT00335153). The study methodology has been reported on^22^ and is summarized below. The study protocol was approved by the institutional review board/ethics committee at all 86 centers in 16 countries. All patients provided written informed consent.

### Study Design

**Fig. 1:**
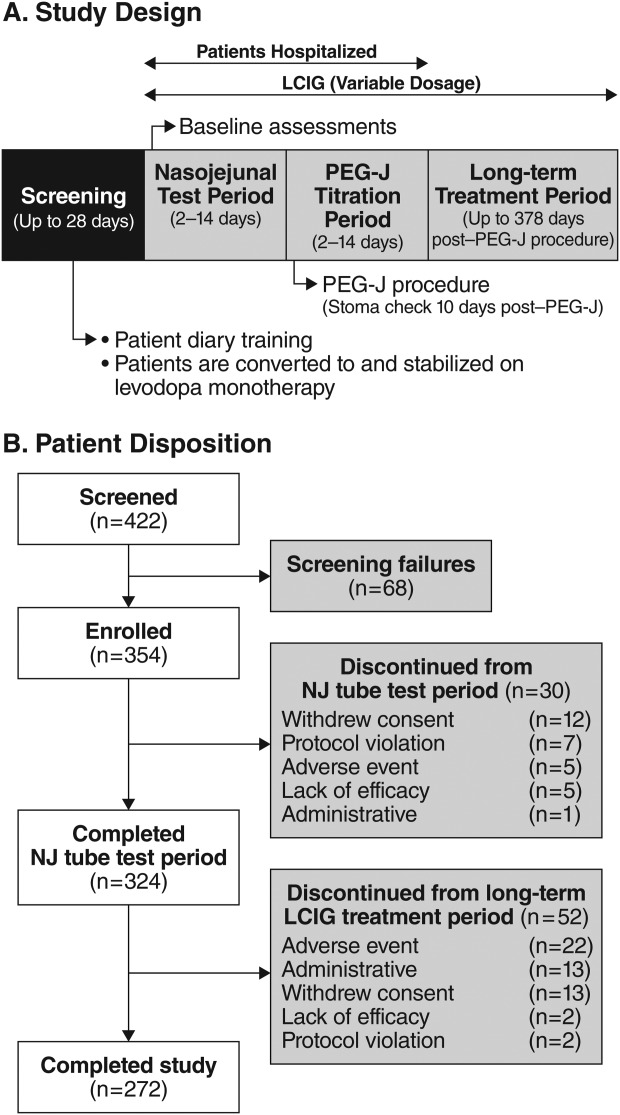
(A) Study design. (B) Patient disposition.

The study included a screening period (≤28 days), baseline assessments, a nasojejunal (NJ) titration period (2-14 days), a PEG-J titration period (2-14 days), and a 54-week treatment period (Fig. 1A). The starting infusion dose was based on each patient's previous daily dose of oral l-dopa. Usage of other PD medications that required tapering off was compensated for at the investigator's discretion. Patients were hospitalized for NJ tube placement and initiation of LCIG titration as well as PEG-J tube placement and further dose optimization by PEG-J, if required. At the end of titration, patients entered long-term PEG-J treatment and assessments began on day 28. LCIG was administered by a portable pump during waking hours; a morning dose/bolus was followed by continuous infusion for approximately 16 hours with additional rescue doses during the day, if clinically indicated. The use of oral immediate-release l-dopa-carbidopa was permitted only when the pump was turned off at night. Use of other PD medications was permitted after 28 days post-LCIG initiation at the investigator's discretion. Apomorphine and controlled-release l-dopa-carbidopa were not permitted.

Safety and tolerability provided the primary endpoint whereas efficacy assessments provided the secondary endpoints.

### Patients

Eligible patients were ≥30 years old, l-dopa responsive, met UK Parkinson's Disease Society Brain Bank diagnostic criteria, and had severe motor fluctuations defined as ≥3 hours of daily off time at baseline (confirmed by the PD symptom diary), despite optimized treatment with available PD medications.

### Safety

Safety measures included AEs, infusion device complications, and tolerability assessed by number of patients completing the study. Laboratory results, vital signs, and electrocardiogram (ECG) were monitored.

AEs were coded according to Medical Dictionary for Regulatory Activities (MedDRA) version 14.0. Each event could be coded to one or more terms descriptive of the event. Planned hospitalization for baseline assessment and treatment initiation was not considered a serious AE (SAE) unless hospitalization was prolonged as a result of complications. All AEs reported are treatment-emergent AEs, which were defined as those that began or worsened from the time of NJ tube insertion until 30 days after PEG-J removal. AEs of special interest were monitored. These were AEs associated with neuropathy, the procedure and device (e.g., PEG-J placement), respiratory tract aspiration, weight loss, and cardiovascular fatalities.

### Efficacy

Efficacy outcomes included assessed mean change from baseline to last visit in patient-diary off time, “on” time with troublesome dyskinesia, and on time without troublesome dyskinesia (on time without dyskinesia plus on time with nontroublesome dyskinesia, i.e., does not interfere with function or cause meaningful discomfort); the investigator-rated Clinical Global Impression-Improvement (CGI-I) scale; the UPDRS^23^ Parts II, III, total score (Parts I–III), and the dyskinesia items from Part IV (questions 32-34); the 39-item PD Questionnaire (PDQ-39)^24^; the EuroQoL-5D (EQ-5D) Summary Index^25^; and the EuroQoL visual analog scale (EQ-VAS).^26^ Efficacy assessments were collected during post-PEG-J weeks 4, 12, 24, and 54; PD diary and CGI-I were also collected at post-PEG-J week 36. PDQ-39 was administered during screening visit 1 rather than at baseline.

Patients were trained to record their motor states every 30 minutes throughout the waking day using a 24-hour diary (Hauser diary)^27^ over the 3 consecutive days preceding baseline and each scheduled visit. PD diary variables were normalized to a 16-hour waking day and averaged over the 3 consecutive days. At baseline, clinicians rated the severity of patients' symptoms with the Clinical Global Impression-Severity (CGI-S) scale. During treatment, clinicians used the CGI-I scale. The UPDRS was administered by the investigator during the best on state (usually 2-4 hours after the morning dose).

### Statistical Analyses

Efficacy analyses included all patients who received LCIG during the post-PEG-J period and completed ≥1 postbaseline efficacy assessment. Safety analysis included all patients who had NJ placement and completed ≥1 postbaseline safety evaluation. The within-group magnitude of change for all efficacy outcome measures was tested using a one-sample *t* test. Multiple testing procedures were not used to control for study-wise type I error rate. Planned enrollment was 320 patients to provide a sufficient sample to satisfy regulatory requirements for exposure assessments at 6 and 12 months.

## Results

### Patient Disposition and Baseline Measures

Of 354 enrolled patients, 324 (91.5%) completed the NJ phase and 272 completed the study (76.8% of all patients enrolled, 84.0% of those who proceeded to PEG-J treatment; Fig. 1B).

Eighty-two patients (23.2%) prematurely discontinued, of whom 27 (7.6%) discontinued as the result of an AE. Other reasons for withdrawal were administrative reasons (4.0%; e.g., protocol-specified discontinuations for timely study closure), major protocol violations (2.5%), lack of efficacy (2.0%), and withdrawal of consent (7.1%; reasons for withdrawal of consent were not collected).

At baseline, patients had a mean ± standard deviation (SD) age of 64.1 ± 9.1 years, PD duration of 12.5 ± 5.5 years, and off time of 6.75 ± 2.35 hours per day (Table 1). Ninety-four patients (26.6%) were on l-dopa (or l-dopa derivative) monotherapy, whereas 259 (73.2%) were receiving ≥2 PD medications (including l-dopa for all patients) in any combination (primarily dopamine agonists [55.4% of all patients], amantadine [29.9%], and catechol-*O*-methyltransferase (COMT) inhibitors [28.2%]), all of which were discontinued before LCIG treatment.

**TABLE 1:** Baseline characteristics (n = 354)

Characteristic	Value
Age, years	
Mean ± SD	64.1 ± 9.1
Sex, males, n (%)	202 (57.1)
Race, n (%)	
White	328 (92.7)
Asian	22 (6.2)
Black	4 (1.1)
Weight, kg	
Mean ± SD	70.8 ± 15.8
Median (range)	69.5 (39.7-123.0)
PD duration in years, mean ± SD	12.5 ± 5.5
l-dopa dose at screening, mg/day, mean ± SD	1,082.9 ± 582.1
PD medications, n (%)^a^	
Number of PD medication classes received	
One (all l-dopa or derivative alone)	94 (26.6)
Two	112 (31.6)
Three	87 (24.6)
More than three	60 (16.9)
Medication classes of those receiving ≥2 PD medications	
l-dopa or derivatives	259 (73.2)
Dopamine agonists	196 (55.4)
COMT inhibitors	100 (28.2)
Amantadine	106 (29.9)
MAO-B inhibitors	45 (12.7)
Tertiary amines	11 (3.1)
Not recorded	1 (0.3)
Off time in hours/day,^b^ mean ± SD	6.75 ± 2.35
On time without troublesome dyskinesia in hours/day,^b^ mean ± SD	7.65 ± 2.45
On time with troublesome dyskinesia in hours/day,^b^ mean ± SD	1.61 ± 2.03
CGI-S scale,^b^,^c^ mean ± SD	4.85 ± 0.84
UPDRS scores,^d^ mean ± SD	
Total (sum of Parts I, II and III)^e^	48.4 ± 18.9
Part II (activities of daily living)^f^	17.4 ± 6.6
Part III (motor symptoms)^g^	28.8 ± 13.7
Part IV (dyskinesia items nos. 32, 33 and 34 only)^e^	3.7 ± 2.4
PDQ-39 Summary Index score,^h^ mean ± SD	42.8 ± 15.1
EQ-5D Summary Index score,^i^ mean ± SD	0.588 ± 0.195
EQ-VAS score,^i^ mean ± SD	50.2 ± 21.0

a Some patients' medications were tapered and discontinued before baseline; listed drug categories are those used by ≥3.0% of all patients.

b n = 316.

c The CGI-S is a 7-point Likert scale ranging from 1 (normal) to 7 (most ill).

d Higher UPDRS scores are associated with more disability.

e n = 292.

f n = 293.

g n = 291.

h n = 320; Higher PDQ-39 scores are associated with more severe symptoms.

i n = 318; Higher EQ scores are associated with better health.

### Total Daily l-dopa Dose

All patients were converted to l-dopa-carbidopa monotherapy based on the l-dopa component of their previous PD therapy. On the last titration day (NJ or PEG-J, whichever was a patient's final titration period), the mean total daily l-dopa dose was 1,547.4 mg, which included a mean of 1,537.0 mg from LCIG; only 4.4% (15 of 338) of patients received oral immediate-release l-dopa-carbidopa at nighttime, with an average dose of 235 mg. Initial titration during the NJ period was completed in a mean of 4.5 ± 2.2 days. The mean LCIG dose remained relatively constant throughout the study, ranging from 1,551.0 to 1,630.5 mg, depending on time point, and was 1,572.4 mg at last visit (Supporting Fig. 1). In the post-PEG-J phase, 76.5% (n = 248) of patients received only l-dopa-carbidopa, as LCIG with or without oral l-dopa-carbidopa, including 27.8% (n = 90) who received LCIG monotherapy. Among patients receiving adjunctive medications, 12.7% (n = 41) received dopamine agonists, 9.6% (n = 31) amantadine, 3.7% (n = 12) COMT inhibitors, and 1.5% (n = 5) monoamine oxidase (MAO)-B inhibitors (Supporting Table 1). At last visit, 27.8% (n = 88) of patients received immediate-release l-dopa-carbidopa (mean total dosage: 174.6 mg/night).

### Safety

AEs were reported in 166 (46.9%) patients during the NJ period; the most common AEs were insomnia (7.9%), complication of device insertion (7.3%), and oropharyngeal pain (6.5%). During the post-PEG-J period, 298 (92.0%) patients experienced AEs (Table 2). The most common were complication of device insertion (34.9%), abdominal pain (31.2%), and procedural pain (20.7%). For the majority of subjects, AEs were mild (18.5%) or moderate (43.8%) and transient, with the highest incidence occurring during week 1 post-PEG-J (Supporting Fig. 2), with 65.1% of patients experiencing an AE at week 1 post-PEG-J, compared with 24.4%, 15.4%, and 17.1% by weeks 2, 3, and 4, respectively. SAEs were reported in 105 (32.4%) patients; the most common included complication of device insertion (6.5%), abdominal pain (3.1%), and peritonitis and polyneuropathy (each 2.8%; Table 2). There were no clinically meaningful changes in laboratory values, vital signs, or ECG.

**Fig. 2:**
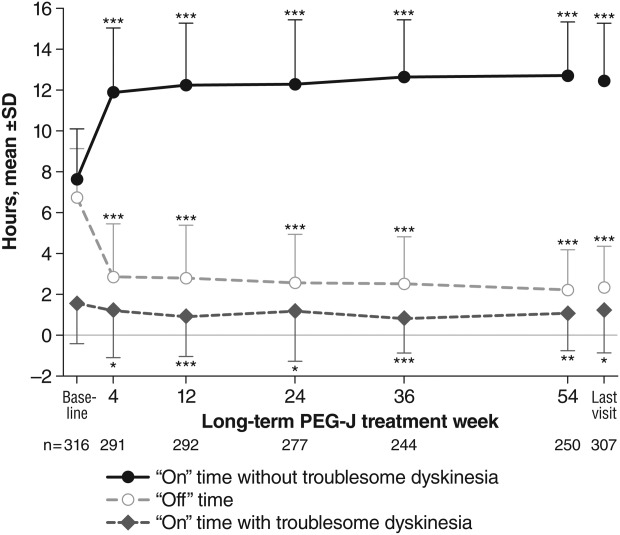
Mean ± SD daily “off” and “on” times as assessed by a Parkinson's disease diary. *P < 0.05; ^**^P < 0.01; ^***^P<0.001 versus baseline.

**TABLE 2:** AEs and SAEs in the percutaneous endoscopic gastrojejunostomy treatment period (n = 324)

MedDRA Preferred Term^a^	No. of Patients (%)
Any AE	298 (92.0)
AEs reported in ≥10%	
Complication of device insertion^b^	113 (34.9)
Abdominal pain	101 (31.2)
Procedural pain	67 (20.7)
Nausea	54 (16.7)
Excessive granulation tissue	52 (16.0)
Postoperative wound infection	50 (15.4)
Fall	49 (15.1)
Constipation	47 (14.5)
Insomnia	44 (13.6)
Incision site erythema	42 (13.0)
Urinary tract infection	37 (11.4)
Any SAE	105 (32.4)
SAEs reported in ≥1%	
Complication of device insertion^b^	21 (6.5)
Abdominal pain	10 (3.1)
Peritonitis	9 (2.8)
Polyneuropathy	9 (2.8)
PD^c^	8 (2.5)
Pneumoperitoneum	8 (2.5)
Hip fracture	6 (1.9)
Pneumonia	6 (1.9)
Device dislocation	5 (1.5)
Depression	4 (1.2)

a A single event could be coded to >1 preferred term.

b Events with this term were most often additionally coded to abdominal pain, abdominal discomfort, abdominal distension, flatulence, and pneumoperitoneum.

c Patients requiring hospitalization or extended hospitalization resulting from PD.

Among AEs of special interest, procedure-/device-related AEs were reported for 68.5% of patients, primarily complication of device insertion (33.6%), abdominal pain (26.5%), procedural pain (20.4%), excessive granulation tissue (15.4%), postoperative wound infection (15.1%), incision-site erythema (12.7%), procedural-site reaction (9.3%), postprocedural discharge (7.7%), incision-site pain (6.2%), and pneumoperitoneum (5.9%). There were no treatment-emergent cardiovascular fatalities. Aspiration-related AEs (14.8% of patients) were primarily dyspnea (4.0%), pneumonia (3.1%), gastroesophageal reflux disease (2.2%), pyrexia (2.2%), dysphagia (1.9%), and atelectasis (1.5%). Fourteen aspiration events occurred within 7 days of initial PEG placement and 3 within 7 days of tube replacement/repositioning that required endoscopy. AEs related to polyneuropathy (6.8% of patients) were coded to the following MedDRA preferred terms: polyneuropathy (3.1% [which led to discontinuation for 1 patient]); peripheral sensory neuropathy (0.9%); Guillain-Barré syndrome-like neuropathy (coded as Guillain-Barré Syndrome; see Discussion; 0.6%); mononeuropathy (0.6%); neuralgia (0.6%); neuropathy peripheral (0.6%); and peripheral sensorimotor neuropathy (0.6%). Weight-loss–related AEs occurred in 15.4% of patients.

Twenty-seven (7.6%) patients had an AE leading to withdrawal, 5 during the NJ period, and 22 patients post-PEG-J. Withdrawals during the NJ period were the result of dysphagia, vomiting, and complication of device insertion in 1 patient as well as pneumonia, QT prolongation, anxiety, and hallucination (1 patient each). In the post-PEG-J period, the most common reasons were complication of device insertion (n = 6), abdominal pain (n = 3), dyskinesia (n = 2), death of unknown etiology (n = 2), and completed suicide (n = 2; both patients had a history of depression). There were 8 subjects who had procedure-/device-related AEs resulting in discontinuation.

A total of 8 deaths (2.3%) were reported; none were considered treatment related. Seven of these deaths occurred during the LCIG treatment period or within 30 days after PEG-J removal and included deaths attributed to suicide (n = 2), unknown etiology (n = 2), multiple complications (n = 1), cerebrovascular accident (n = 1), and cachexia (n = 1). One patient with a history of deep vein thrombosis (DVT) died 93 days post-PEG-J removal (i.e., not treatment emergent) as a result of DVT.

Device complications (i.e., related to device function, but not necessarily associated with an AE) were reported for 87.0% of patients: intestinal tube complication (50.9%); pump or stoma complication (35.8% each); and PEG-J or other complication (35.2% each).

### Efficacy

Off time was significantly decreased from baseline to last visit by 4.4 ± 2.9 hours per day, or 65.6% (*P* < 0.001; Fig. 2 and Supporting Table 2). This improvement was sustained throughout all post-PEG-J visits (weeks 4-54; *P* < 0.001). Similarly, on time without troublesome dyskinesia increased by 4.8 ± 3.4 hours per day, or 62.9% (*P* < 0.001), and on time with troublesome dyskinesia decreased by 0.4 ± 2.8 hours per day, or 22.5% (*P* = 0.023). These improvements were sustained at all visits (*P* < 0.05).

On the CGI-I scale at end of treatment, 22.4% of patients were “very much improved,” 55.5% “much improved,” and 13.7% “minimally improved.” There was no change for 3.1% of patients, whereas 2.8% were “minimally worse,” 1.0% “much worse,” and none were “very much worse.”

**Fig. 3:**
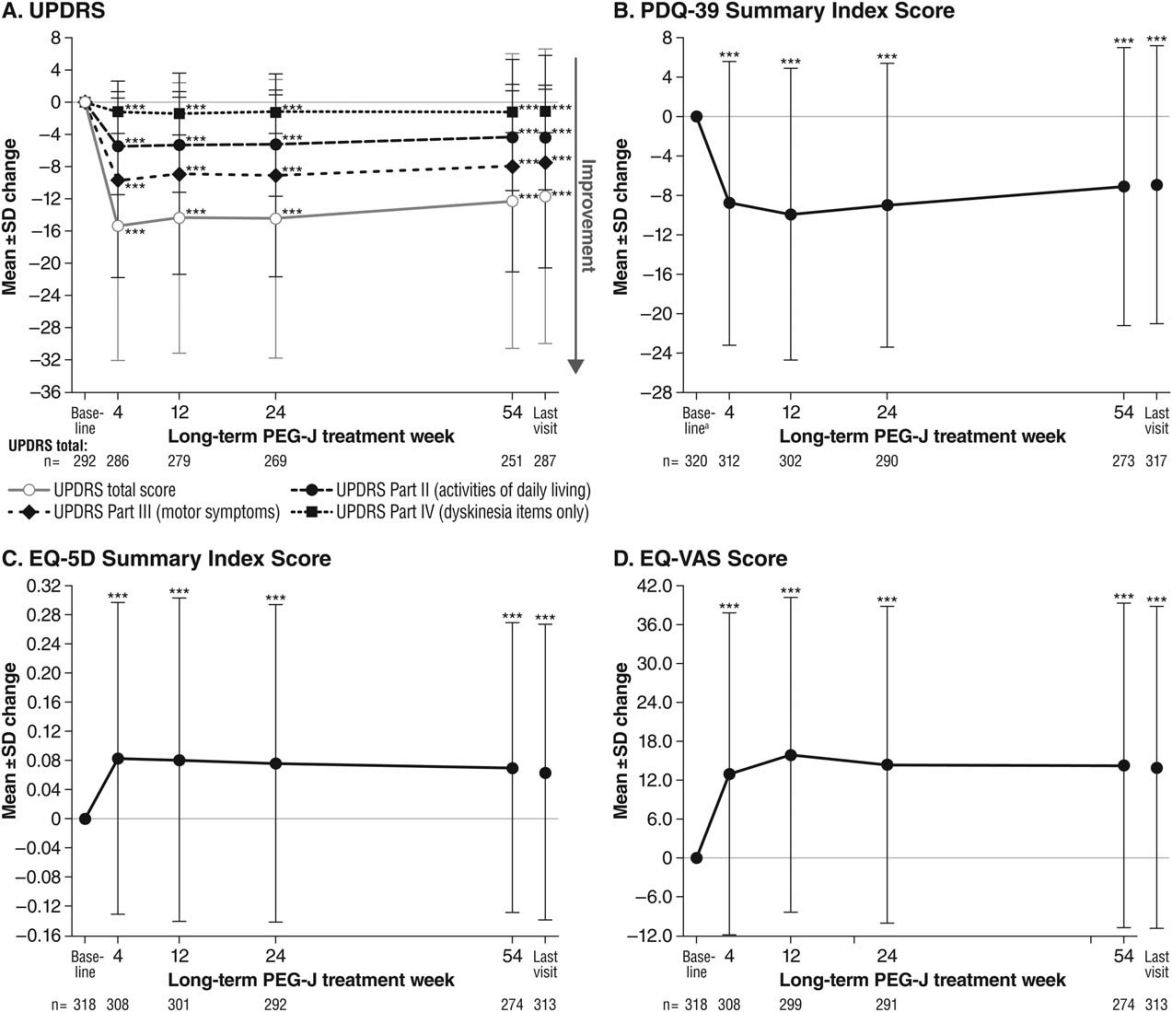
Mean ± SD changes from baseline on other efficacy measures including function and health-related quality of life. ^a^Baseline value from screening. ^***^P < 0.001 versus baseline, one-sample *t* test.

On the efficacy measures commonly associated with function and health-related quality of life (HRQoL), significant improvement (*P* < 0.001) was noted by week 4 of long-term treatment and was maintained through the end of the study (Fig. 3). From baseline to last visit, the mean change was −4.4 ± 6.5 points for the UPDRS Part II (activities of daily living), +0.064 ±0.203 points for the EQ-5D Summary Index, and +14.0 ± 24.8 points for the EQ-VAS. From screening to last visit, the mean ± SD change in the PDQ-39 Summary Index was −6.9 ± 14.1 points. Seven of the eight PDQ-39 domains (except social support) showed statistically significant mean improvements (Supporting Table 3).

## Discussion

This prospective study provides long-term safety and efficacy data for over 12 months in the largest cohort to date of patients with advanced PD treated with LCIG. Here, LCIG was initiated as monotherapy, replacing both oral l-dopa and other adjunctive PD medications in patients with PD who experienced severe motor complications despite optimized pharmacological therapy. Continuous infusion of LCIG throughout the day led to significant improvements in off time of −4.4 hours per day (65.6%), as assessed by patient-completed diary, which were sustained throughout the 54-week trial. This outcome is of a magnitude expected to be clinically meaningful to patients, well beyond the 1-hour change in off time deemed clinically important in the literature.^28^ Of note, the reduction in off time corresponded to a significant increase in on time without troublesome dyskinesia. Even with optimized LCIG, there was some residual off time (approximately 2.5 hours in this cohort), but both the physician- and patient-perceived improvements were robust, with significant and enduring improvements in motor function as assessed by the UPDRS and CGI-I, as well as HRQoL as assessed by the PDQ-39 and EuroQoL. In fact, HRQoL improvements began as early as week 4 and were maintained through the duration of the 54-week study period. Furthermore, total daily dosing, after initial titration/optimization, was stable throughout the study, suggesting that patients do not develop tolerance to LCIG. Moreover, although adjunctive therapies were permitted after 28 days, there was low use of these therapies and 76.5% of patients remained on l-dopa-carbidopa monotherapy. This is valuable given that it simplifies patient treatment regimens and could decrease AEs resulting from multiple dopaminergic medications.

The most common AEs in this study were associated with device insertion, were generally transient, and decreased substantially after the first week post-PEG-J tube placement. Device complications were most common in the first week after PEG-J placement. In the NJ phase, insomnia may have been related to causes including hospitalization itself and was deemed not related to the system in the majority of cases. SAEs occurred in 105 (32.4%) patients; the most common included complication of device insertion (6.5%), abdominal pain (3.1%), and peritonitis and polyneuropathy (each 2.8%). There were 2 SAEs of suicide, both in subjects under the age of 65 with a medical history of depression; neither reported suicidal ideation, but patients with PD as a group are at increased risk of suicide (by 5.3-fold in one study),^29^ and clinically relevant depression has been reported in 35% of patients with PD.^30^ In a study employing multivariable regression, the only factor associated with suicidal ideation or behavior in advanced PD was severity of depression,^31^ so physicians should be vigilant about the emotional state of all patients with advanced PD. Considering the patient population in our study (mean 64.1 years old, mean PD duration of 12.5 years; baseline CGI-S of “markedly ill” or worse for approximately two thirds), the procedure was generally well tolerated with few discontinuations resulting from AEs (7.6%). Of the 272 subjects who completed this study, 203 (74.6%) enrolled in the extension study, 66 (24.3%) transitioned to commercial LCIG, and 3 (1.1%) discontinued treatment.

To further examine procedure-related AEs, an adjudication committee consisting of independent expert gastroenterologists reviewed treatment-emergent AEs and SAEs categorized as “procedure and device-associated events” in the ongoing LCIG phase III program, including this study. The committee found that the rate of gastrointestinal AEs was generally consistent with ranges reported in the literature^32^,^33^ for the PEG-J procedure.^34^

Patients with PD are at increased risk of neuropathy. The cause of this is uncertain, but it has been suggested that it may be related to the metabolic effects of long-term l-dopa therapy.^35^ The rate in our study is consistent with reports in the literature for patients with PD receiving l-dopa.^36^ In the LCIG phase III program, an independent committee adjudicated cases associated with polyneuropathy and determined that the cases observed were predominantly subacute or chronic, the severity mild to moderate, the phenotype sensory or sensorimotor, and the neurophysiology was typically consistent with axonal polyneuropathy.^37^ No patients were deemed to fulfill the criteria for Guillain-Barré syndrome despite 2 AEs being coded as Guillain-Barré syndrome. Although neuropathy screening and monitoring was not standardized at the outset, decreased vitamin B_6_, vitamin B_12_, folic acid, and increased homocysteine and methylmalonic acid appear to have emerged as risk factors for polyneuropathy,^36^,^38^ and measurements at baseline and every 3 months were added by amendment to the LCIG phase III protocols. Future prospective studies examining the incidence of neuropathy would be valuable.

The open-label design of this trial and lack of a control group are study limitations, in that the potential contributions of placebo effect cannot be assessed. However, the present trial is the largest sample of LCIG-treated patients studied worldwide thus far, which is a key strength. Moreover, these results demonstrate the maintenance of LCIG effects over 12 months, which is consistent with the recently reported 12-week, double-blind, double-dummy, phase III study comparing LCIG with optimized oral immediate-release l-dopa-carbidopa (both treatments concomitant with unchanged adjunctive therapies).^15^ The double-blind study showed that the difference in off time decrease was significant at −1.91 hours (*P* = 0.0015; least squares [LS] mean of −4.04 hours for LCIG [n = 35] vs. −2.14 hours for oral l-dopa-carbidopa [n = 31] over an “optimized” baseline). Also in line with our study, the median CGI-I endpoint score was “much improved” for LCIG versus “minimally improved” for oral therapy, the mean UPDRS Part II score changed by −1.8 points (LS mean) in the LCIG arm (3.0-point improvement over oral therapy; *P* = 0.0086), and the PDQ-39 Summary Index score changed by −10.9 points (LS mean) with LCIG (7-point improvement over oral therapy; *P* = 0.0155).

Safety results in the double-blind study were consistent with our study. The most common AEs were related to the procedure, device, oral l-dopa, or underlying disease, most commonly abdominal pain (42%), procedural pain (32%), and nausea (25%). AEs were generally mild to moderate and declined within the first 2 weeks following the PEG-J procedure. Gastrointestinal AEs were typical for a PEG-J procedure.

Continuous drug delivery is integral to the therapeutic profile of LCIG. In countries where it is approved, LCIG is indicated for the treatment of advanced l-dopa-responsive PD with severe motor fluctuations and dyskinesia when available combinations of oral PD medications have not given satisfactory results.^39^ In this setting, the restricted mean duration of LCIG treatment in Sweden was approximately 7.8 years; 60% of patients were ongoing, and the most common reason for discontinuation was death (unrelated to LCIG).^40^ Furthermore, when LCIG is initiated, the large majority of patients do not require adjunctive agents and can be maintained on l-dopa-carbidopa monotherapy, facilitating dose adjustment for efficacy or managing AEs^14^ and simplifying patients' therapeutic regimens. Overall, our safety and efficacy results are further reinforced by results from these and other LCIG studies that have been systematically compiled and published.^41^

Apomorphine infusion and DBS are also associated with significant reductions in off time and with HRQoL improvements.^13^,^42^–^50^ LCIG will provide another treatment option for patients with motor complications despite optimized therapy, offering an additional treatment option suiting patient-specific needs and contraindications.^50^

In summary, in this long-term, open-label study, LCIG demonstrated sustained, significant, and clinically meaningful improvements not only in motor complications, but also in HRQoL in advanced PD. LCIG was associated with robust improvements in off and on time, at a consistent mean daily dose throughout the study period, and without worsening dyskinesia throughout 54 weeks. As assessed by the low rate of study withdrawal resulting from AEs (7.6%), LCIG was generally well tolerated. Nonetheless, 92% of the patients reported ≥1 AE, most commonly associated with PEG-J tube placement during the first week post-PEG-J placement. LCIG provides an effective therapeutic option for advanced PD patients with severe motor complications despite optimized oral pharmacologic therapy.

## Study Investigators

The authors thank the following study investigators for their contributions to this study: Prof. Giovanni Abbruzzese (Universita degli Studi di Genova, Genova, Italy); Alina Agafina, MD, PhD (St. Petersburg City Hospital, St. Petersburg, Russia); Tim Anderson, FRACP, MD (University of Otago, Christchurch, New Zealand); Roongroj Bhidayasiri, MD, FRCP (King Chulalongkom Memorial Hospital, Bangkok, Thailand); Prof. Andrzej Bogucki, MD, PhD (Medical University of Lodz, Lodz, Poland); Prof. Ubaldo Bonuccelli (University of Pisa, Pisa, Italy); James T. Boyd, MD (University of Vermont College of Medicine, Burlington, VT, USA); Dr. Roger Clarnette (Fremantle Hospital, Fremantle, Australia); Dr. Luis Cunha (Hospital da Universidade de Coimbra, Coimbra, Portugal); Dr. Oriol de Fàbregues (Universitat Autonoma de Barcelona, Barcelona, Spain); Prof. Dr. Günther Deuschl (Kiel University, Kiel, Germany); Dirk Dressler, MD, PhD (Hannover Medical School, Hannover, Germany); Andrew Feigin, MD (The Feinstein Institute for Medical Research, Manhasset, NY, USA); Prof. Joaquim J. Ferreira, MD, PhD (Instituto de Medicina Molecular, Lisbon, Portugal); Tanya Gurevich, MD (Tel Aviv Medical Center, Tel Aviv, Israel); Jonathan Harris, MD (Neurologic Consultants, Ft. Lauderdale, FL, USA); Neal Hermanowicz, MD (University of California Irvine, Irvine, CA, USA); Dr. Donald S. Higgins, Jr. (Albany Medical College, Albany, NY, USA); Dr. Andrew Hughes (Austin Health, Melbourne, Australia); Keith L. Hull, Jr., MD (Wake Med Health and Hospitals at Raleigh, Raleigh, NC, USA); Dr. Zygmunt Jamrozik (Samodzielny Publiczny Centralny Szpital, Warsaw, Poland); Thomas E. Kimber, MBBS (Hons), PhD (Royal Adelaide Hospital, Adelaide, Australia); Jaime Kulisevsky, MD, PhD (Sant Pau Hospital, Universitat Autònoma de Barcelona, Barcelona, Spain); Rajeev Kumar, MD (Colorado Neurological Institute, Englewood, CO, USA); Laura Kupila, MD, PhD (Päijät-Häme Central Hospital, Lahti, Finland); Prof. Patricia Limousin (Institute of Neurology, London, UK); Michael A. Lobatz, MD (The Neurology Center and Scripps Health, Encinitas, CA, USA); Dr. Zoltan Mari (John Hopkins Neurology, Baltimore, MD, USA); Juan Carlos Martinez-Castrillo, MD, PhD (Hospital Ramon y Cajal, IRYCIS, Madrid, Spain); Leo Verhagen Metman, MD, PhD (Rush University Medical Center, Chicago, IL, USA); Stuart Mossman, MD, FRACP (Wellington Hospital, Wellington, New Zealand); Prof. Thomas Müller (St. Joseph Hospital Berlin-Weißensee, Berlin, Germany); Prof. Giuseppe Orefice (Azienda Ospedaliera Universitaria, Napoli, Italy); Fernando L. Pagan, MD (Georgetown University Hospital, Washington, DC, USA); Nicolas Phielipp, MD (Toronto Western Hospital, Tornonto, Ontario, Canada); Niphon Poungvarin, MD, FRCP (Siriraj Hospital, Bangkok, Thailand); Dr. Victor Puente (Hospital del Mar, Barcelona, Spain); Brad A. Racette, MD (Washington University School of Medicine, St. Louis, MO, USA); Prof. Ivan Rektor, MD, CSc (Masaryk University, Hospital Sv. Anna, CEITEC, Brno, Czech Republic); Ramon L. Rodriguez, MD (University of Florida, Gainesville, FL, USA); Maria Jose Rosas, MD (Hospital S. João, Porto, Portugal); Evzen Ruzicka, MD, DSc (Vseobecna Fakultnin Nemocnice Praha, Praha, Czech Republic); Joseph M. Savitt, MD, PhD (Parkinson's and Movement Disorders Center of Maryland, Elkridge, MD, USA); Johannes Schwarz, MD (Geriatric Hospital Haag, Haag, Germany); Mustafa S. Siddiqui, MD (Wake Forest University School of Medicine, Winston-Salem, NC, USA); Barry J. Snow, MBChB, FRACP, FRCPC (Auckland City Hospital, Auckland, New Zealand); Martin Sommer, MD (University of Goettingen, Goettingen, Germany); David G. Standaert, MD, PhD (University of Alabama at Birmingham, Birmingham, AL, USA); Fabrizio Stocchi, MD, PhD (IRCCS San Raffaele, Rome, Italy); Oksana Suchowersky, MD, FRCPC (University of Alberta, Edmonton, Alberta, Canada); Paul L. Timmings, MBChB, MD, FRACP (Waikato Hospital, Hamilton, New Zealand); Prof. Eduardo Tolosa (Universitat de Barcelona, Barcelona, Spain); Associate Prof. Martin Vališ, MD, PhD (Charles University, Faculty Hospital, Hradec Kralove, Czech Republic); C.C.P. Verstappen, PhD, MD (Canisius Wilhelmina Hospital, Nijmegen, The Netherlands); Thomas Vogt, MD (University Medical Center, Mainz, Germany); Richard Walsh, MD (Toronto Western Hospital, Toronto, Ontario, Canada); Cheryl Waters, MD (Columbia University, New York, NY, USA); William J. Weiner, MD† (University of Maryland, Baltimore, MD); Christian Winkler, MD, PhD (University Hospital Freiburg, Freiburg, Germany); Jaroslaw Wronka, MD (NZOZ CMHCP Oddzial Neurologiczny, Poznan, Poland); Prof. Eduard Yakupov (LLC Research Medical Complex, Kazan, Russia); Cindy Zadikoff, MD (Northwestern University, Chicago, IL, USA); Mario Zappia, MD (University of Catania, Catania, Italy); Katerina Zarubova, MD (Fakultni Nemocnice Motol, Praha, Czech Republic); and Richard M. Zweig, MD (LSU Health Sciences Center, Shreveport, LA, USA). †29 December 2012.

## Supporting Data

Supplementary Information Table 3.

Supplementary Figure 1. Mean total daily dose of levodopa (LCIG plus immediate-release levodopa). SD, standard deviation; NJ, nasojejunal; PEG-J, percutaneous endoscopic gastrojejunostomy.

Supplementary Figure 2. Incidence of AEs (≥10% during any time interval) over time. A single event could be coded to >1 preferred term. NJ, nasojejunal; PEG-J, percutaneous endoscopic gastrojejunostomy.
