# Supplementary material for: Levodopa-Carbidopa Intestinal Gel in Advanced Parkinson'd Disease: Final 12-Month, Open-Label Results
Source: Mov Disord. 2014 Dec 24;30(4):500–9. doi: 10.1002/mds.26123 (PMC4674978; doi:10.1002/mds.26123)
Supplement: Supplementary file 1 — Supplementary Information Table 1. [file mds0030-0500-sd1.docx]

**Supplementary Table 1.** Concomitant PD medications to LCIG during the post–PEG-J treatment period (n=324)

| **Concomitant medication** | **n (%)** |
| --- | --- |
| No concomitant PD medication | 90 (27.8) |
| Levodopa-carbidopa only | 158 (48.8) |
|  |  |
| Levodopa or derivatives | 219 (67.6) |
| Dopamine agonists  Amantadine | 41 (12.7)  31 (9.6) |
| COMT inhibitors  MAO-B inhibitors | 12 (3.7)  5 (1.5) |
| Number of concomitant PD medication classes received |  |
| One | 169 (52.2) |
| Two | 55 (17.0) |
| Three or more | 10 (3.1) |

PD, Parkinson disease; LCIG, levodopa-carbidopa intestinal gel; PEG-J, percutaneous endoscopic gastrojejunostomy; COMT, catechol-*O*-methyltransferase; MAO-B, monoamine oxidase-B.
