# Supplementary material for: Levodopa-Carbidopa Intestinal Gel in Advanced Parkinson'd Disease: Final 12-Month, Open-Label Results
Source: Mov Disord. 2014 Dec 24;30(4):500–9. doi: 10.1002/mds.26123 (PMC4674978; doi:10.1002/mds.26123)
Supplement: Supplementary file 2 — Supplementary Information Table 2. [file mds0030-0500-sd2.docx]

**Supplementary Table 2.** Parkinson’s disease diary and Clinical Global Impression efficacy endpoints

|  | **Baseline** | **Week 4** | **Week 12** | **Week 24** | **Week 36** | **Week 54** | **Last Visit** |
| --- | --- | --- | --- | --- | --- | --- | --- |
| Diary results, hours/day, mean ±SD | (n=316) | (n=291) | (n=292) | (n=277) | (n=244) | (n=250) | (n=307) |
| “Off” time | 6.75 ±2.35 | 2.88 ±2.58 | 2.82 ±2.54 | 2.55 ±2.37 | 2.53 ±2.29 | 2.21 ±1.97 | 2.32  ±2.05 |
| “On” time without troublesome dyskinesia | 7.65 ±2.45 | 11.85 ±3.16 | 12.20 ±3.05 | 12.24 ±3.20 | 12.64 ±2.78 | 12.69 ±2.63 | 12.44 ±2.79 |
| “On” time with troublesome dyskinesia | 1.61 ±2.03 | 1.26 ±2.35 | 0.98 ±2.04 | 1.21 ±2.48 | 0.84 ±1.71 | 1.11 ±1.88 | 1.24  ±2.10 |
| Clinical Global Impression^a^ scale, mean ±SD | (n=316)  4.85 ±0.84 | (n=309)  2.18 ±0.89 | (n=302)  2.21 ±0.96 | (n=291)  2.07 ±0.90 | Not measured | (n=271)  1.99 ±0.83 | (n=316)  2.10  ±0.95 |

Weeks are post–PEG-J procedure.

^a^Clinical Global Impression-Severity scale score at baseline (the 7-point scale ranges from 1 [normal] to 7 [most ill]); Clinical Global Impression-Improvement scale score at all other time points (the 7-point scale ranges from 1 [very much improved] to 7 [very much worse]).
