# Supplementary material for: Levodopa-Carbidopa Intestinal Gel in Advanced Parkinson'd Disease: Final 12-Month, Open-Label Results
Source: Mov Disord. 2014 Dec 24;30(4):500–9. doi: 10.1002/mds.26123 (PMC4674978; doi:10.1002/mds.26123)
Supplement: Supplementary file 3 — Supplementary Information Table 3. [file mds0030-0500-sd3.docx]

**Supplementary Table 3.** PDQ-39 Summary Index and subdomain score (n=317)

| **Variable** | **Score, mean ±SD** | | **Score change,**  **mean ±SD** |
| --- | --- | --- | --- |
|  | **Screening visit 1** | **Last visit** |  |
| Summary Index  Mobility  ADL  Emotional well-being  Stigma  Social support  Cognition  Communication  Bodily discomfort | 42.7 ±15.0  58.8 ±22.8  50.7 ±22.3  39.4 ±21.8  32.5 ±26.0  17.2^a^ ±19.7  27.2 ±18.6  34.3 ±20.4  46.2 ±22.9 | 35.8 ±16.8  47.6 ±26.0  42.4 ±24.3  35.2 ±20.6  23.3 ±22.9  16.9^a^ ±20.0  22.7 ±17.3  30.4 ±22.1  40.5 ±23.0 | –6.9 ±14.1***  –11.2 ±23.4***  –8.3 ±22.6***  –4.2 ±19.7***  –9.1 ±22.1***  –0.3^a^ ±18.9  –4.5 ±18.0***  –3.9 ±19.4***  –5.8 ±22.4*** |

****P*<0.001, one-sample *t* test.

^a^n=315.

PDQ-39, Parkinson’s Disease Questionnaire; SD, standard deviation.
